# Home Treatment of Childhood Illnesses Prior to Seeking Formal Care: A Cross‐Sectional Study at Different Health Care Levels in Ghana

**DOI:** 10.1111/tmi.70042

**Published:** 2025-09-25

**Authors:** Lydia Helen Rautman, Felix Osei Boateng, Isaac Darko Agyiri, Ebenezer Ahenkan, Jones Ankomah, Asare Baffour, Maike Maria Lamshöft, Nicole S. Struck, Jürgen May, Oumou Maiga‐Ascofaré, Ralf Krumkamp

**Affiliations:** ^1^ Department of Infectious Disease Epidemiology Bernhard Nocht Institute for Tropical Medicine Hamburg Germany; ^2^ University Medical Center Hamburg‐Eppendorf Hamburg Germany; ^3^ German Center for Infection Research, Hamburg‐Borstel‐Lübeck‐Riems Braunschweig Germany; ^4^ Infectious Disease Epidemiology Research Group, Kumasi Centre for Collaborative Research in Tropical Medicine Kumasi Ghana; ^5^ Presbyterian Hospital Agogo (PreHA) Agogo Ghana; ^6^ St. Francis Xavier Hospital (SFXH) Assin Foso Ghana; ^7^ Department of Pharmacology Kwame Nkrumah University of Science and Technology Kumasi Ghana

**Keywords:** antibiotic use, Ghana, home treatment, paediatric care

## Abstract

**Background:**

Many caregivers in Sub‐Saharan Africa attempt to manage childhood illnesses at home, which can delay or complicate later diagnosis and treatment at a health facility. Understanding home treatment practices among children could help characterise treatment history when information is unavailable or unreliable. We investigated these practices among children seeking care at three levels of a healthcare system in Ghana.

**Methods:**

Children under 15 years of age and their caregivers were recruited from Community‐based Health Planning and Services, outpatient departments and inpatient departments in Agogo and Assin Foso, Ghana. Demographic, clinical, socioeconomic and home treatment data were collected via interviews. Urine samples from children were tested for antibiotic use. Hierarchical log‐binomial regression models were calculated to estimate risk ratios and control for confounding.

**Results:**

Caregivers of 1503 children were interviewed. Forty‐six percent (*n* = 689) reported any home treatment prior to the visit: 37% (*n* = 560) reported antipyretic use, 11% (*n* = 167) antimalarial use and 7% (*n* = 103) antibiotic use. Home medication was lower at Community‐based Health Planning and Services (30%, *n*/*N* = 148/500) compared to the outpatient departments (61%, *n*/*N* = 308/509) and inpatient departments (47%, *n*/*N* = 233/494). Children treated at home had longer delays in seeking treatment (median 3 days, IQR: 1, 3) compared to those not treated at home (median 2 days, IQR: 1, 3). In regression models, illness severity and specific symptoms were more strongly associated with antimalarial use than with antibiotic use. For most samples where antibiotic inhibition was detected, no prior antibiotic use had been reported (*n*/*N* = 33/46), indicating undisclosed or unrecognised antibiotic intake.

**Conclusions:**

The discrepancy between self‐reported antibiotic use and antibiotic inhibition suggests a lack of awareness about medication identification and appropriate use. This presents a challenge for clinicians in obtaining an accurate treatment history, which is highly relevant to the timely diagnosis and treatment of the illness in the facility.

**Abbreviations**
CG: caregiver
CHPS: Community Health and Planning Services
DHS: Demographic and Health Surveys
HIV: human immunodeficiency virus
IPD: inpatient department
IQR: interquartile range
LRTI: lower respiratory tract infection
MIS: malaria indicator survey
OPD: outpatient department
PR: prevalence ratio
PreHA: Presbyterian Hospital Agogo
RDT: rapid diagnostic test
SES: socioeconomic status
SFXH: Saint Francis Xavier Hospital, Assin Foso
URTI: upper respiratory tract infection
UTI: urinary tract infection

## 1 Introduction

Infectious diseases remain among the top contributors to childhood mortality in Sub‐Saharan Africa [1, 2]. Medications for these diseases are available, but delays in seeking health care and lacking diagnostic tools make it challenging to quickly and effectively treat the illnesses. In many low‐resource areas, long travel and waiting times, high treatment costs and inconsistent medication availability often motivate caregivers to attempt to manage the illness at home with medications or herbal treatments before seeking formal care, that is, from a clinic or health facility [3, 4, 5].

In Ghana, medications are available from several sources, including health clinics, pharmacies/chemists, over‐the‐counter medicine sellers and herbalists, and families also report sharing and using medications leftover from a previous illness [6]. National guidelines mandate that antimalarial medication be dispensed only after a positive malaria rapid test or microscopic demonstration of parasites, yet inappropriate use has been documented in health facilities [7, 8] and in the community, where antimalarials are sold over‐the‐counter in informal medicine outlets [9]. A few antibiotics are authorised for non‐prescription dispensing if recommended by a trained pharmacist [10]. All other antibiotics require a prescription but in reality are frequently dispensed without one if there is client demand [6, 10, 11] and can be sold on credit or in partial doses if a client can't afford the entire regimen [6, 12]. Studies in the region have described the frequent use and misuse of antimicrobials in the home management of illness episodes [3, 4, 6, 13, 14, 15]. When formal care is eventually sought, prior medication use can result in false negative test results, potentially delaying diagnosis and treatment [16]. Inappropriate application or incorrect dose of antimicrobials can also facilitate the development of resistances [17], which with time could result in challenges in treating childhood illnesses if first‐line drugs become ineffective and reserved drugs are unavailable.

Previous studies have examined treatment practices within the context of the healthcare system. However, data on self‐medication practices and their influence on care‐seeking behaviours, especially across different healthcare facilities, remain limited. This study offers novel insights by characterising the prevalence and type of exposure to home treatment among children seeking care at three distinct levels of health care settings. A further combination of self‐reported antibiotic use with objective measures provides unique insights that show the true extent of self‐medication and its impact on healthcare delivery.

## 2 Methods

The study was conducted in a malaria‐endemic area of Ghana where transmission peaks during the rainy season. Patients were recruited at St. Francis Xavier Hospital (SFXH) in Assin Foso, Central Region and Presbyterian Hospital Agogo (PreHA), Ashanti Region. Both hospitals serve the respective peri‐urban population as well as the more rural neighbouring communities. In both Agogo and Assin Foso, children were recruited at the local paediatric outpatient department (OPD), inpatient department (IPD) and a Community Health Planning and Services (CHPS) compound, for a total of six sites. CHPS provides standard childhood immunisations and primary healthcare for more rural populations and can dispense antimalarial and antibiotic medication; more severe cases are referred to the nearest hospital.

Based on a prior study of hospitalised children at the PreHA [2], we conservatively estimated a prevalence of self‐medication with antimicrobials (antibiotic or antimalarial) in 10% of patients on the ward, 5% at the OPD and 1% at CHPS. With a power of 80%, 435 participants per stratum were required to sufficiently detect these differences at this level. To account for possible missing data and loss to follow‐up, we aimed to recruit a total of 1500 total participants to our study.

Recruitment was done during the rainy season, from late April to August 2024, where malaria prevalence was expected to be high. Inclusion criteria were child age between 30 days and 15 years, and the accompanying adult was required to be older than 18 years and the primary caretaker of the child. Children seeking care at the facility for the first time for the current illness were eligible for recruitment, and children presenting with conditions such as burns, trauma, injury, wounds and allergic reactions were excluded.

Structured interviews were conducted by trained interviewers to collect information on demographics, clinical data including symptoms and perceived severity, home treatment practices and socioeconomic details. Interview data was collected directly with the REDCap mobile application. After the initial clinical examination according to the standard of care, test results, suspected diagnosis and prescriptions were recorded. At all sites, malaria rapid diagnostic tests (RDTs) were used to diagnose malaria among suspected cases, typically febrile patients. Microscopy was also available at hospitals as an alternative diagnostic or to confirm positive malaria RDTs and diagnose hyperparasitemia if requested by the treating physician. As part of the standard of care, rapid tests for other relevant infections (i.e., urinary tract infection (UTI), typhoid fever, human immunodeficiency virus (HIV), gonorrhoea, hepatitis B) were available at all sites except the Agogo CHPS compound, where patients with conditions other than uncomplicated malaria were diagnosed clinically or referred to the PreHA. At the OPD and IPD sites, full blood counts could be done and bacterial cultures were available.

Urine samples were collected where possible from children after the interview and prior to administration of medication at the health facility. The urine sample was transferred to the lab and inoculated with *Bacillus subtilis* , a bacterial strain highly sensitive to antibiotics. The culture was incubated for 18–36 h to screen for antibiotics; if no growth was detected, antibiotic inhibition was reported. The outcome of the visit was recorded from electronic health records of the clinics at 2 weeks post‐interview.

Socioeconomic variables were used to design a score using principal component analysis; scores for observations with missing values were imputed by random forest using the *missForest* package in *R* [18]. Scores were dichotomised to ‘low’ for the lower and ‘high’ for the higher mathematical half of possible scores. Hierarchical log‐binomial regression models with mixed effects were used for complete observations to estimate crude (bivariable regression) and adjusted (multivariable regression) prevalence ratios (PRs) of different factors for home antimalarial and antibiotic use with site and location as a second‐level predictor using the *lme4* and *logbin* packages in *R*. All analyses were conducted and graphics produced in *R* (version 4.3.0) [19].

## 3 Results

### 3.1 Description of the Study Group

**FIGURE 1:**
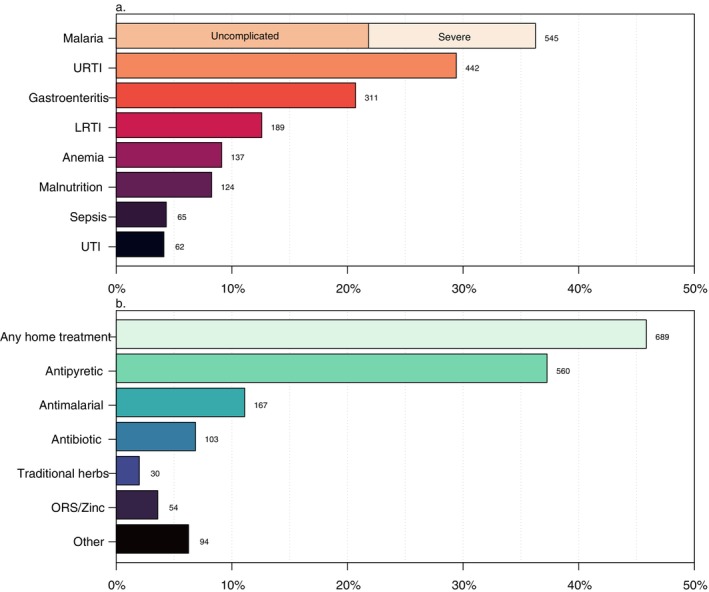
Frequency and prevalence of (a) suspected diagnoses and (b) home treatment strategies among 1503 children. LRTI, lower respiratory tract infection; ORS, oral rehydration solution; URTI, upper respiratory tract infection; UTI, urinary tract infection.

The caregivers of 1503 children participated in the study. Forty‐seven percent (*n* = 709) of the study children were female and the median age was 3.4 years (IQR = 1.6, 6.6) (Table 1). The most common symptoms were fever (79%, *n* = 1186), headache (37%, *n* = 550), cough (35%, *n* = 528), vomiting (28%, *n* = 418) and abdominal pain (25%, *n* = 381). Convulsions were more frequently reported in the IPD (12%, *n*/*N* = 61/494) compared to the OPD (3%, *n*/*N* = 17/509) and CHPS (1%, *n*/*N* = 6/500), as was difficulty breathing (15%, *n* = 75 in the IPD compared to 4%, *n* = 21 in the OPD and 1%, *n* = 5 at CHPS). Thirty‐eight percent (*n*/*N* = 389/1029) of children tested positive with a malaria RDT. The most common suspected diagnoses were malaria (36%), upper respiratory tract infection (URTI) (29%) and gastroenteritis (21%) (Figure 1a). Caregivers' perception of the child's illness was most often mild at CHPS (51%, *n*/*N* = 255/500), moderate at the OPD (69%, *n*/*N* = 351/509) and severe in the IPD (49%, *n*/*N* = 243/494). Sixty percent (*n* = 299) of families at CHPS had a low SES score, compared to 27% (*n* = 136) at the OPD and 45% (*n* = 220) at the IPD. Median transport time to the health facility was 15 min (IQR 10, 30).

**TABLE 1:** Distribution of characteristics by health facility type.

	Total	CHPS	OPD	IPD
*n*	1503 (100%)	500 (33%)	509 (34%)	494 (33%)
Female sex of the child	709 (47%)	258 (52%)	229 (45%)	222 (45%)
Health insurance	1380 (92%)	447 (89%)	494 (97%)	439 (89%)
Child's median age in years (IQR)	3.4 (1.6, 6.6)	3.7 (1.9, 7.2)	3.0 (1.4, 6.0)	3.4 (1.5, 6.3)
Median days since symptom start (IQR)	2.0 (1.0, 3.0)	2.0 (1.0, 3.0)	2.0 (1.0, 3.0)	2.0 (1.0, 3.0)
Perceived mild illness	434 (29%)	255 (51%)	96 (19%)	83 (17%)
Perceived moderate illness	731 (49%)	213 (43%)	351 (69%)	167 (34%)
Perceived severe illness	336 (22%)	32 (6%)	61 (12%)	243 (49%)
Treatment sought within 48 h of symptom start	560 (37%)	170 (34%)	209 (41%)	181 (37%)
Median travel time in minutes (IQR)	15.0 (10.0, 30.0)	15.0 (9.5, 30.0)	15.0 (10.0, 30.0)	19.0 (10.0, 40.0)
Travel time > 60 min	42 (3%)	4 (1%)	14 (3%)	24 (5%)
Low SES score	655 (44%)	299 (60%)	136 (27%)	220 (45%)
Malaria RDT positive	389/1029 (38%)	183/445 (41%)	60/264 (23%)	146/320 (46%)
Malaria microscopy positive	242/610 (40%)	49/76 (65%)	62/256 (24%)	131/278 (47%)
Antibiotic inhibition	46/490 (9%)	9/235 (4%)	18/171 (11%)	19/84 (23%)

*Note*: ‘Travel time’ refers to the reported length of time the caregiver and child spent travelling from their home to the chosen health facility.

Abbreviations: CHPS, community health and planning services; IPD, inpatient department; IQR, interquartile range; OPD, outpatient department; RDT, rapid diagnostic test; SES, socioeconomic status.

### 3.2 Time From Symptom Start to Seeking Treatment

Symptoms started a median of 2 days (IQR = 1, 3) prior to the visit. This was 3 days (IQR = 1, 3) for children whose caregivers perceived their illness as mild, 2 days (IQR = 1, 3) for moderate and 2 days (IQR = 1, 3) for severe illness. Prior home treatment was reported for more than half (56%, *n*/*N* = 351/626) of children brought to a facility more than 48 h after symptom start, compared to 38% (*n*/*N* = 335/872) among children for whom care was sought within 48 h. There was no clear relationship between age and time duration of symptoms prior to seeking treatment.

### 3.3 Home Treatment Prior to the Current Visit

Forty‐six percent (*n* = 689) of all caregivers reported having treated the child at home prior to the current visit: the most common reported medications were antipyretics 37% (*n* = 560), antimalarials 11% (*n* = 167) and antibiotics 7% (*n* = 103) (Figure 1b). The prevalence of any home treatment was lowest at CHPS (30%, *n* = 148) and highest in the OPD (61%, *n* = 308) compared to the IPD (47%, *n* = 233). One‐quarter (25%, *n*/*N* = 41/165) of antimalarials and 20% of antibiotics (*n*/*N* = 20/102) used at home were sourced from the informal sector, including having been purchased from a drug seller, left over from a previous illness, shared or other. The rest were sourced from CHPS or a chemist/pharmacy.

Twenty‐five percent (*n*/*N* = 42/167) of caregivers who treated with antimalarials had done an RDT at home and 37% (*n*/*N* = 48/131) discontinued using antimalarials after less than 3 days prior to seeking health care at the hospital or CHPS compound. More than half (56%, *n*/*N* = 27/48) of those who discontinued using antimalarials early were diagnosed with malaria at the health facility, compared to 44% (*n*/*N* = 42/95) of those who used antimalarials for at least 3 days. Among children who had taken antimalarials, use for the correct duration (3 days) was more frequent among those who had received their antimalarials from CHPS (83%, *n*/*N* = 43/52) compared to a chemist/pharmacy (44%, *n*/*N* = 21/48) or informal sources (drug or medicine seller, left over, shared or other; 35%, *n*/*N* = 10/29). Half (51%, *n*/*N* = 86/167) of children who were treated with an antimalarial at home were not diagnosed with malaria during the visit.

**FIGURE 2:**
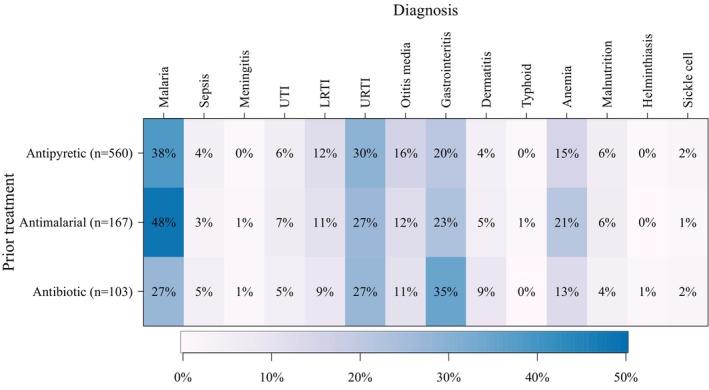
Heatmap of suspected diagnoses by reported prior treatment. Prevalences given are calculated as the percent of children in each treatment group with the diagnosis. LRTI, lower respiratory tract infection; URTI, upper respiratory tract infection; UTI, urinary tract infection.

Most frequent diagnoses for children who took antipyretic medication prior to the visit were malaria (38%, *n*/*N* = 214/560), URTI (30%, *n*/*N* = 165/560) and gastroenteritis (21%, *n*/*N* = 115/560) (Figure 2). This was similar for patients with prior antimalarial use (49% malaria, *n*/*N* = 81/167; URTI 27%, *n*/*N* = 45/167; gastroenteritis 23%, *n*/*N* = 39/167), along with anaemia (21%, *n*/*N* = 35/167). For children with prior antibiotic use, gastroenteritis was the most common diagnosis (35%, *n*/*N* = 36/103), followed by URTI (27%, *n*/*N* = 28/103) and malaria (27%, *n*/N = 28/103).

**FIGURE 3:**
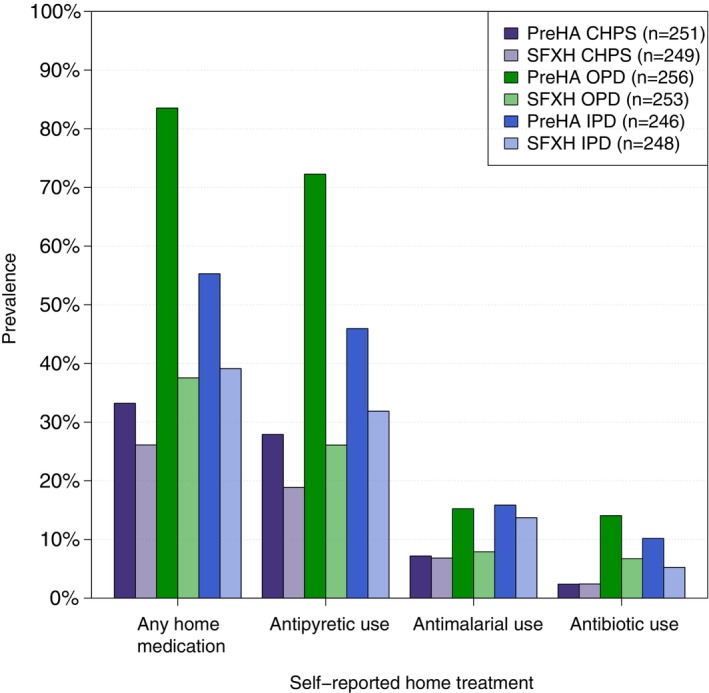
Prevalence of home treatment practices by site and location. CHPS, Community Health Planning and Services; IPD, inpatient department; OPD, outpatient department; PreHA, Presbyterian Hospital Agogo; SFXH, St. Francis Xavier Hospital Assin Foso.

Home treatment was more frequent in Agogo compared to Assin Foso for antipyretic (49%, *n*/*N* = 368/753 vs. 26%, *n*/*N* = 192/750), antibiotic (9%, *n* = 67 vs. 5%, *n* = 36) and antimalarial treatment (13%, *n* = 96 vs. 9%, *n* = 71). This was consistent across sites but especially prominent in the OPD (Figure 3).

### 3.4 Antibiotic Inhibition

Urine samples were successfully collected in approximately one‐third of patients (*n*/*N* = 490/1503). Inhibition was demonstrated among 9% (*n*/*N* = 46/490) of samples and was most common among children at the IPD (23%, *n*/*N* = 19/84) compared to the OPD (11%, *n*/*N* = 18/171) and CHPS (4%, *n*/*N* = 9/235). Of all children with antibiotic inhibition, caregivers of 33 of 46 did not report having given an antibiotic prior to inhibition. Antibiotic inhibition was more frequent in Assin Foso (12%, *n*/*N* = 37/298) compared to Agogo (5%, *n*/*N* = 9/192), as was the lack of reported antibiotic use among positive samples (84%, *n*/*N* = 31/37 in Assin Foso vs. 22%, *n*/*N* = 2/9 in Agogo).

### 3.5 Outcomes

For children admitted to the ward, the median length of hospital stay was 3 days (IQR: 2, 5). This was 3.5 days (IQR: 3, 6) in children reporting prior antibiotic use and 3 days (IQR: 2, 5) in children who did not. The length of stay was similar in children who did and did not report antimalarial use (for both, median 3 days, IQR: 2, 5). Two children visiting the Assin Foso IPD died during follow‐up. One child was diagnosed with severe malaria and meningitis and reported prior antipyretic use; the other was diagnosed with anaemia and URTI and reported prior antipyretic and antimalarial use for a recent malaria infection.

### 3.6 Regression Models

**FIGURE 4:**
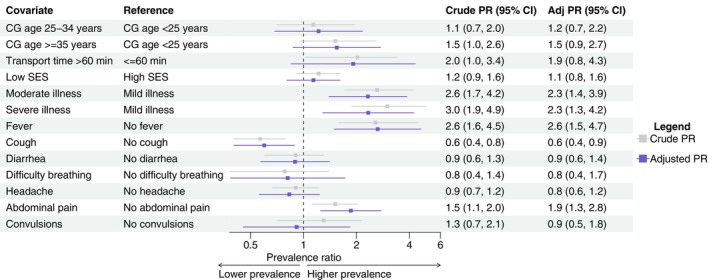
Hierarchical log‐binomial regression model for antimalarial use with site and location as second‐level predictor. Adj, adjusted; CG, caregiver; PR, prevalence ratio; SES, socioeconomic status. The reference group is individuals with no antimalarial or antibiotic use.

**FIGURE 5:**
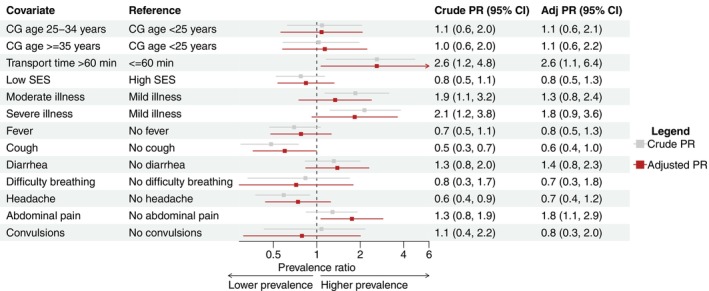
Hierarchical log‐binomial regression model for antibiotic use with site and location as second‐level predictor. Adj, adjusted; CG, caregiver; PR, prevalence ratio; SES, socioeconomic status. The reference group is individuals with no antimalarial or antibiotic use.

Hierarchical log‐binomial regression models were used to estimate crude and adjusted prevalence ratios (aPR) for different factors on antimalarial and antibiotic use. In the antimalarial model, prevalence was higher for moderate (aPR: 2.3, 95% CI: 1.4, 3.9) and severe illness (aPR: 2.3, 95% CI: 1.3, 4.2) compared to mild illness, as well as for fever (aPR: 2.6, 95% CI: 1.5, 4.7) and abdominal pain (aPR: 1.9, 95% CI: 1.3, 2.8) (Figure 4). Prevalence was lower among those reporting cough (aPR: 0.6, 95% CI: 0.4, 0.9). In the antibiotic use model, prevalence was higher among those with a transport time > 60 min (aPR: 2.6, 95% CI: 1.1, 6.4) and those reporting abdominal pain (aPR: 1.8, 95% CI: 1.1, 2.9) (Figure 5).

## 4 Discussion

Home treatment of childhood illnesses prior to seeking care is common in areas of Sub‐Saharan Africa where medications are easily accessible and geographical and financial barriers to seeking formal care are prominent. Our findings provided insights on the case and treatment histories of children seeking care at three levels of the healthcare system in Ghana.

### 4.1 Home Treatment Practices

Exact estimates of prevalence of home treatment in Ghana vary based on the region and study design. The 2019 Ghana MIS estimated that around 40% of families who sought care for a febrile child < 5 years in the 2 weeks prior to the survey did so at a pharmacy or drug store instead of attending a health facility [20]. One longitudinal household study in Ghana estimated the prevalence of self‐medication at 70% for illness episodes [6] and another study of hospitalised children in Agogo reported 76%, although this was mostly antipyretic use [2]. Prevalence of home treatment in our study (approximately half of participants) was somewhat lower, but we observed that care was sought later for children with reported prior treatment, supporting other studies with similar findings [21, 22].

In our study, caregivers that perceived the illness as moderate or severe sought treatment faster than those who thought the illness was mild, suggesting the influence of severity on the decision to seek formal care. Caregivers attempted to identify and treat malaria cases at home, as evidenced by the home use of RDTs and antimalarial medication, although in many cases the treatment was inappropriate or incorrectly applied, especially when obtained from somewhere other than a health facility.

Higher prevalence ratios of antimalarial use among children with specific symptoms including fever, abdominal pain and lack of cough, and more severe perceived illness indicate the caregiver's consideration of the child's state and symptoms in the decision to treat with antimalarials at home. Furthermore, for antibiotic use, an increased prevalence ratio was associated with greater travel time to a facility, and a lack of increased prevalence among children with specific symptoms (aside from abdominal pain) or illness severity suggests that antibiotics are used more indiscriminately at home and when geographic barriers are especially high.

Clearly, caregivers are motivated to identify and manage mild childhood illnesses on their own but lack medical expertise and understanding of appropriate treatment practices, which can result in the inappropriate use of medications as seen in our studies as well as in the literature [3, 6, 15]. Community education initiatives to improve recognition of symptoms and increase the capacity to apply over‐the‐counter medications could improve outcomes of mild illness and ensure early treatment‐seeking for more serious conditions.

### 4.2 Differences Between Facilities

Differences in home treatment practices between levels of healthcare had, prior to this study, not been assessed. Because of the design of our study, the facility sites were more a measure of illness severity than deliberate presentation to a specific type of facility, especially for the IPD, since most children presented at the OPD before being admitted. Overall, medication use prior to the visit was less frequent at CHPS compared to the OPD and IPD. CHPS serves a more rural and poorer population; thus, home treatment use could be less common among these families because of the cost of medication or lower availability in rural areas.

### 4.3 Antibiotic Inhibition

Reported antibiotic treatment at home was relatively uncommon, but the very low reporting of antibiotic treatment among children whose urine demonstrated antibiotic inhibition suggests that self‐reports substantially underestimate actual home treatment practices. This could be due to a social desirability bias or lack of knowledge about which medications are antibiotics. This indicates that self‐reported antibiotic use in this study, and likely other studies in the region, is strongly underestimated, although estimating the degree to which bias may impact self‐reporting may be a challenge. Alternative strategies, such as presenting the caregivers with a sample of common medication packages and asking them to indicate which was used, may improve the accuracy of this reporting. To address the root of this issue, education initiatives about the types and uses of different medications could also improve caregiver understanding of and ability to identify commonly used antibiotics.

### 4.4 Medication Sourcing

Significant medication sourcing from the informal sector was reported in our study. These estimates (although likely an underestimate) were similar to those of the 2019 DHS MIS for Ghana [20], supporting concerns surrounding the use of low‐quality drugs and inadequate drug seller training that have been observed elsewhere [12]. In our study, antimalarials were taken for less than the standard regimen of 3 days [23] more often when obtained from the informal sector compared to those obtained from CHPS. Due to the range of possible antibiotic regimen durations, we were unable to assess this for antibiotics, but similar behaviour could present a significant issue and contribute to the development of antibiotic resistances. Since regulation enforcement is unlikely to change [24] and informal drug outlets will continue to be motivated to sell antibiotics by client demand and financial incentives [10, 11], communities should be the focus of education programmes and other intervention efforts to reduce non‐prescription antibiotic use.

### 4.5 Implications of Home Treatment

Prior medication use was reported for many children, which can complicate diagnosis at the health facility. Clinicians should collect data on the type and duration of prior medication taken, as an incomplete course of medication may not entirely clear an infection. Malaria RDTs can also remain positive for weeks after antimalarial treatment, so the diagnosis of the co‐infection causing the ongoing symptoms could be delayed if the clinician isn't made aware of the prior use of antimalarials and instead diagnoses malaria as the cause of symptoms [25]. Home antibiotic use can result in false negative culture results, potentially delaying diagnosis and treatment [16]. For some children, the need to seek professional care after failed home treatment could indicate a severe or resistant infection, so obtaining an accurate treatment history will be crucial in ensuring fast and appropriate treatment.

### 4.6 Limitations

Our study was designed to be informative about the treatment practices of those with access to the healthcare system and those whose attempts at home treatment failed, making it necessary to seek formal care. It is probable that many more children had an infection that was successfully managed at home or cleared without the use of medication; this may include up to 31% of children with fever, according to the 2019 Malaria Indicator Survey (MIS) in Ghana [20]. Therefore, conclusions must be interpreted with this in mind and studies that aim to assess the prevalence and nature of all home treatment practices must be designed to recruit families in non‐health facility contexts.

Due to logistical challenges, urine sampling was lower in Agogo than in Assin Foso. Urine sampling was low in the IPD because patients were frequently given antibiotics prior to recruitment, making them ineligible for antibiotic inhibition testing. Furthermore, providing a urine sample was voluntary and the collection of a urine sample was challenging for very young children; thus, better estimates of antibiotic inhibition prevalence in younger age groups may require adaptations to the study protocol.

Despite our efforts to create a study with comparable sites, differences in the prevalence of home treatment between Agogo and Assin Foso reduced the exchangeability of the sites, with a higher prevalence of prior medication use in Agogo across the board. These differences were most prominent between the OPDs and IPDs compared to CHPS, suggesting significant behavioural differences between the lower (CHPS) and upper (OPD and IPD) levels of the healthcare system. Clearly, treatment practices can be highly localised, so conclusions about practices by location are not broadly generalisable and must be interpreted with caution. Finally, our study was conducted during the rainy season; during the dry season, illness aetiologies and burden may differ [26, 27], impacting home use of antimicrobials and model outputs [28].

## 5 Conclusion

Our findings demonstrated the misidentification of and confusion surrounding the appropriate use and duration of medications, many of which were obtained without the oversight of a trained medical professional. These practices can hinder the accurate diagnosis and successful treatment of childhood illnesses when formal care is eventually sought. Our findings highlight community education initiatives on illness recognition, use of over‐the‐counter medications, and appropriate care‐seeking behaviours as potentially important tools in minimising unnecessary medication use, improving the effectiveness of healthcare delivery, and achieving better health outcomes.

## Ethics Statement

This study and informed consent procedures were approved by the Committee on Human Research, Publications and Ethics, School of Medical Science, Kwame Nkrumah University of Science and Technology, Kumasi, Ghana (CHRPE/AP/179/24).

## Consent

Prior to enrollment, written informed consent was obtained from the primary caregiver of the participant for both their participation in the interview and on behalf of the child.

## Conflicts of Interest

The authors declare no conflicts of interest.

## Data Availability

De‐identified data and analysis code are available for download from Zenodo, an open‐access data repository, at https://doi.org/10.5281/zenodo.15412218.

## References

[1] UNICEF W, World Bank, and UN Population Division , Levels & Trends in Child Mortality ‐ Report 2023 (World Health Organization, 2023).

[2] L. H. Rautman , O. Maiga‐Ascofaré , D. Eibach , et al., “Fever in Focus: Symptoms, Diagnoses and Treatment of Febrile Children in Ghana—A Longitudinal Hospital Study,” Tropical Medicine & International Health 29, no. 3 (2024): 206–213. 38093593 10.1111/tmi.13962

[3] M. Ocan , E. A. Obuku , F. Bwanga , et al., “Household Antimicrobial Self‐Medication: A Systematic Review and Meta‐Analysis of the Burden, Risk Factors and Outcomes in Developing Countries,” BMC Public Health 15, no. 1 (2015): 1–11. 26231758 10.1186/s12889-015-2109-3 PMC4522083

[4] J. Nonvignon , M. K. Aikins , M. A. Chinbuah , et al., “Treatment Choices for Fevers in Children Under‐Five Years in a Rural Ghanaian District,” Malaria Journal 9, no. 1 (2010): 1–8. 20584280 10.1186/1475-2875-9-188 PMC2914057

[5] T. Kassile , R. Lokina , P. Mujinja , and B. P. Mmbando , “Determinants of Delay in Care Seeking Among Children Under Five With Fever in Dodoma Region, Central Tanzania: A Cross‐Sectional Study,” Malaria Journal 13 (2014): 1–10. 25182432 10.1186/1475-2875-13-348 PMC4159519

[6] M. A. Ahiabu , P. Magnussen , I. C. Bygbjerg , and B. P. Tersbøl , “Treatment Practices of Households and Antibiotic Dispensing in Medicine Outlets in Developing Countries: The Case of Ghana,” Research in Social & Administrative Pharmacy 14, no. 12 (2018): 1180–1188. 29428578 10.1016/j.sapharm.2018.01.013

[7] H. A. Bonful , A. K. Awua , M. Adjuik , et al., “Extent of Inappropriate Prescription of Artemisinin and Anti‐Malarial Injections to Febrile Outpatients, a Cross‐Sectional Analytic Survey in the Greater Accra Region, Ghana,” Malaria Journal 18, no. 1 (2019): 1–11. 31558149 10.1186/s12936-019-2967-8 PMC6764136

[8] N. Y. Boadu , J. Amuasi , D. Ansong , E. Einsiedel , D. Menon , and S. K. Yanow , “Challenges With Implementing Malaria Rapid Diagnostic Tests at Primary Care Facilities in a Ghanaian District: A Qualitative Study,” Malaria Journal 15 (2016): 1–12. 26921263 10.1186/s12936-016-1174-0 PMC4769585

[9] S. Ameh , P. Welaga , C. W. Kabiru , et al., “Factors Associated With Appropriate Home Management of Uncomplicated Malaria in Children in Kassena‐Nankana District of Ghana and Implications for Community Case Management of Childhood Illness: A Cross‐Sectional Study,” BMC Public Health 15 (2015): 1–10. 25934315 10.1186/s12889-015-1777-3 PMC4429811

[10] S. Afari‐Asiedu , J. Kinsman , E. Boamah‐Kaali , et al., “To Sell or Not to Sell; the Differences Between Regulatory and Community Demands Regarding Access to Antibiotics in Rural Ghana,” Journal of Pharmaceutical Policy and Practice 11, no. 1 (2018): 1–10. 30564369 10.1186/s40545-018-0158-6 PMC6293650

[11] S. A. Belachew , L. Hall , and L. A. Selvey , “Non‐Prescription Dispensing of Antibiotic Agents Among Community Drug Retail Outlets in Sub‐Saharan African Countries: A Systematic Review and Meta‐Analysis,” Antimicrobial Resistance and Infection Control 10, no. 1 (2021): 1–15. 33446266 10.1186/s13756-020-00880-w PMC7807893

[12] S. O. Bekoe , M. A. Ahiabu , E. Orman , et al., “Exposure of Consumers to Substandard Antibiotics From Selected Authorised and Unauthorised Medicine Sales Outlets in Ghana,” Tropical Medicine & International Health 25, no. 8 (2020): 962–975. 32418294 10.1111/tmi.13442

[13] R. Opoku , B. Dwumfour‐Asare , L. Agrey‐Bluwey , et al., “Prevalence of Self‐Medication in Ghana: A Systematic Review and Meta‐Analysis,” BMJ Open 13 (2023): 64627. 10.1136/bmjopen-2022-064627 PMC10439347 36963791

[14] D. Edessa , N. Assefa , Y. Dessie , F. Asefa , G. Dinsa , and L. Oljira , “Non‐Prescribed Antibiotic Use for Children at Community Levels in Low‐ and Middle‐Income Countries: A Systematic Review and Meta‐Analysis,” Journal of Pharmaceutical Policy and Practice 15, no. 1 (2022): 57. 36180895 10.1186/s40545-022-00454-8 PMC9524137

[15] S. Afari‐Asiedu , M. Hulscher , M. A. Abdulai , E. Boamah‐Kaali , K. P. Asante , and H. F. L. Wertheim , “Every Medicine Is Medicine; Exploring Inappropriate Antibiotic Use at the Community Level in Rural Ghana,” BMC Public Health 20, no. 1 (2020): 1–10. 32664902 10.1186/s12889-020-09204-4 PMC7359511

[16] C. Scheer , C. Fuchs , M. Gründling , et al., “Impact of Antibiotic Administration on Blood Culture Positivity at the Beginning of Sepsis: A Prospective Clinical Cohort Study,” Clinical Microbiology and Infection 25, no. 3 (2019): 326–331. 29879482 10.1016/j.cmi.2018.05.016

[17] World Health Organization , WHO Report on Surveillance of Antibiotic Consumption: 2016–2018 Early Implementation (WHO, 2018).

[18] D. Stekhoven , “missForest: Nonparametric Missing Value Imputation Using Random Forest,” R Package Version 1.5 (2022).

[19] R Studio Team , RStudio: Integrated Development for R (RStudio, PBC, 2020).

[20] Ghana Statistical Service (GSS), ICF , Ghana Malaria Indicator Survey 2019 (GSS and ICF, 2020).

[21] A. Mpimbaza , A. Katahoire , P. J. Rosenthal , C. Karamagi , and G. Ndeezi , “Caregiver Responses and Association With Delayed Care‐Seeking in Children With Uncomplicated and Severe Malaria,” Malaria Journal 17 (2018): 1–12. 30563514 10.1186/s12936-018-2630-9 PMC6299589

[22] M. M. Lamshöft , E. Liheluka , G. Ginski , et al., “Understanding Pre‐Hospital Disease Management of Fever and Diarrhoea in Children—Care Pathways in Rural Tanzania,” Tropical Medicine & International Health 29 (2024): 706–714. 38888511 10.1111/tmi.14022

[23] World Health Organization , Guidelines for the Treatment of Malaria (World Health Organization, 2015).

[24] S. K. Yevutsey , K. O. Buabeng , M. Aikins , et al., “Situational Analysis of Antibiotic Use and Resistance in Ghana: Policy and Regulation,” BMC Public Health 17, no. 1 (2017): 1–7. 29169340 10.1186/s12889-017-4910-7 PMC5701378

[25] T. D. Swarthout , H. Counihan , R. K. K. Senga , and I. Van Den Broek , “Paracheck‐Pf Accuracy and Recently Treated Plasmodium Falciparum Infections: Is There a Risk of Over‐Diagnosis?,” Malaria Journal 6, no. 1 (2007): 1–6. 17506881 10.1186/1475-2875-6-58 PMC1890550

[26] S. M. Adadey , R. Ayee , S. Languon , D. Quansah , and O. Quaye , “Patterns of Frequently Diagnosed Pediatric Morbidities in Hospitalized Children in the Volta Region of Ghana,” Global Pediatric Health 6 (2019): 2333794X19889230. 10.1177/2333794X19889230 PMC6868572 31799337

[27] F. M. Engelaer , D. van Bodegom , J. N. Mangione , U. K. Eriksson , and R. G. Westendorp , “Seasonal Variation in Child and Old‐Age Mortality in Rural Ghana,” Transactions of the Royal Society of Tropical Medicine and Hygiene 108, no. 3 (2014): 147–153. 24473476 10.1093/trstmh/tru007

[28] H. C. Greene , K. Makovi , R. Abdul‐Mumin , A. Bansal , and J. A. Frimpong , “Challenges in the Distribution of Antimicrobial Medications in Community Dispensaries in Accra, Ghana,” PLoS One 19, no. 5 (2024): e0281699. 38809832 10.1371/journal.pone.0281699 PMC11135707

